# Biomass and morphology of fine roots of sugi (*Cryptomeria japonica*) after 3 years of nitrogen fertilization

**DOI:** 10.3389/fpls.2013.00347

**Published:** 2013-09-03

**Authors:** Kyotaro Noguchi, Junko Nagakura, Shinji Kaneko

**Affiliations:** ^1^Shikoku Research Center, Forestry and Forest Products Research InstituteKochi, Japan; ^2^Department of Forest Site Environment, Forestry and Forest Products Research InstituteTsukuba, Japan

**Keywords:** fine-root biomass, fine-root length, fine-root diameter, root tissue density, specific root length, soil depth

## Abstract

Increasing nitrogen (N) deposition may affect carbon and nutrient dynamics in forest ecosystems. To better understand the effects of N deposition, we need to improve our knowledge of N effects on fine roots (roots <2 mm in diameter), as they are a key factor in carbon and nutrient dynamics. In this study, we fertilized 1 × 2 m plots in a sugi (*Cryptomeria japonica*) stand (336 kg ha^-^^1^ y^-^^1^) for 3 years and evaluated the responses of the fine roots to high N load. After fertilization, the concentration of NO_3_–N in the soil of N-fertilized (NF) plots was five-times as large as that in the control plots and the effect was more remarkable in the subsurface soil than in the surface soil. The biomass of fine roots <2 mm in diameter appeared to be greater in the NF plots (88 ± 19 g m^-^^2^) than in the control plots (56 ± 14 g m^-^^2^), but this difference was not statistically significant. In both plots, 76% of the biomass was accounted for by fine roots that were <1 mm in diameter. In the surface soil, the specific root length of fine roots <1 mm in diameter was significantly greater, and the diameter of those fine roots was marginally smaller, in the NF plots than in the control plots. In addition, the concentration of N in fine roots <1 mm in diameter was marginally greater in the NF plots than in the control plots. There may have been increased production of thinner fine roots or increased root branching in the NF plots. This study suggests that, in general, high N load is likely to have positive effects on sugi in terms of fine root characteristics and the effects on fine-root morphology are more evident than the effects on fine-root biomass.

## INTRODUCTION

Fine roots of forest trees are, in general, defined as roots less than 2 mm in diameter. Fine roots are physiologically highly active parts of root systems and play a key role in water and nutrient uptake at the tree–soil interface. In addition, recent studies indicate that fine roots have relatively fast turnover rates and fine root production accounts for a substantial proportion (10–60%) of the net primary production of forests (Hendrick and Pregitzer, 1993; Ostonen et al., 2005). Fine roots are recognized as one of the most important components for tree growth and in the carbon and nutrient dynamics of forest ecosystems.

Nitrogen (N) is quantitatively and functionally the most important nutrient for plants and for other living organisms. Tree roots take up N from the soil in inorganic forms ( ${NH}_{4}^{+}$ and ${NO}_{3}^{-}$; Genenger et al., 2003; Hawkins et al., 2008), while recent studies suggested that organic N compounds, such as amino acids, are also used in many ecosystems (Näsholm et al., 2009). In addition, the amount and spatial distribution of inorganic N in the soil and their chemical forms are likely to affect the biomass and dynamics of fine roots in forest ecosystems (Fujimaki et al., 2004; Tateno et al., 2004).

On the other hand, excessive N can have negative effects on tree growth (Aber et al., 1998). In the past decades, deposition of inorganic N has increased, especially in industrialized countries, and a number of studies have examined the effects of excess N load on forest ecosystems in Europe and the USA. The results of these studies indicate that high N loads can cause the decline of some forests (Boxman et al., 1998; Magill et al., 2004) and increased or unchanged tree growth in other forests (Magill et al., 2004; Nagakura et al., 2006). For example, Magill et al. (2004) showed that 15 years of N addition resulted in a continuous increase in biomass in a hardwood stand, whereas decreased biomass with high mortality was observed in a red pine stand. The varied responses of forest trees to N deposition could be attributed to factors such as the amount of deposition, soil fertility, and tree species characteristics. High levels of N deposition that are related to industrial or other human activity have been observed in East Asia. In Japan, for example, forests close to urban areas received higher levels of N deposition than those in remote areas (Shibata et al., 2001; Yoshinaga et al., 2012). In addition, recent rapid economic growth of some countries, such as China, may cause further increases in N deposition (Liu et al., 2013). Therefore, further studies and monitoring are needed in order to better understand the effects of N deposition on the soil (e.g., the amount and spatial distribution of N, soil acidification, and so on) and tree growth in the forest ecosystems of this region.

Fine roots are physiologically active parts of root systems and their responses to changing environments are relatively quick. Therefore, fine roots can be used as a sensitive indicator of changes in tree physiological status under changing soil environmental conditions, such as drought and acidification (Hirano et al., 2007; Konôpka et al., 2007). Fine roots respond to changes in environmental conditions with changes in fine-root biomass and spatial distribution (Helmisaari et al., 2007; Finér et al., 2011); fine root morphology, including diameter and specific root length (SRL) (Ostonen et al., 2007a, b); and fine root chemistry, such as N content and Al/Ca molar ratio (Hirano et al., 2007; Vanguelova et al., 2007). Improving our knowledge about the responses of fine roots to N fertilization would help us to better understand the effects of the changing N conditions on forest ecosystems.

Recently, a chronic N fertilization experiment was conducted in a 20-year-old sugi (*Cryptomeria japonica*) plantation in eastern Japan (Nagakura et al., 2006). In this experiment, inorganic N was supplied at the rate of 336 kg N ha^-^^1^ y^-^^1^ for 7 years. Although the N concentration in leaves was higher in the fertilized plots during first 3 years of the experiment, there was no significant effect on aboveground growth of the sugi trees. However, another study showed that 3-year N fertilization at the same rate to small plots (1 m × 2 m) significantly affected fine root dynamics (Noguchi et al., 2013). In that study, the elongation rates and residence time of fine roots increased with N fertilization, whereas stem growth was not significantly affected. This suggests that sugi is tolerant of high N loads. Neither of these reports provided information about fine root parameters, such as morphology and N concentration, and further study on these fine root parameters was needed to better understand how the trees could acclimate to high N load. Therefore, the objective of this study was to determine the responses of the fine roots of sugi trees to 3 years of N fertilization in terms of biomass, morphology, and N concentration.

## MATERIALS AND METHODS

### SUGI (*Cryptomeria japonica*)

Sugi is one of the major coniferous species in Japan. Plantations of this species cover half of the plantation area (nearly 20% of all forested area) in the country (Japan FAO Association, 1997) and they are often established on foot slopes or along streams. Rooting depths of sugi trees are about 2–3 m and vertical changes in fine root biomass are moderate (Karizumi, 1974, 1976). In addition, Fujimaki et al. (2007) reported that sugi stands showed higher coefficients for vertical distribution of fine roots (β; Gale and Grigal, 1987) than shallow-rooting species, such as *Chamaecyparis obtusa* and *Picea glauca* (0.95–0.99 for sugi *vs*. 0.91 for *Chamaecyparis obtusa* and 0.89 for *Picea glauca*). Their results suggest that the proportion of fine root biomass in subsoil is larger for sugi than for shallow-rooting species. However, Fujimaki et al. (2007) also found low β values in a young (4-year-old) sugi stand and in a sugi stand that seemed to be N deficient (0.83 and 0.91, respectively), suggesting that the vertical distribution of sugi fine roots are affected by factors such as stand age and soil conditions (Fujimaki et al., 2007). As for symbioses with mycorrhizal fungi, sugi has been reported to form arbuscular mycorrhiza, but not ectomycorrhiza (Matsuda, 1994).

### STUDY SITE

This study was conducted in a 28-year-old sugi (*Cryptomeria japonica*) plantation in eastern Japan (36°10′N, 140°13′E, 41 masl). Mean annual air temperature and mean annual precipitation for 1981–2010 at the Tsuchiura weather station, which is located at 10 km south from the study site (Japan Meteorological Agency, ), were 14.4°C and 1188 mm, respectively. Stand density at the study site was ca. 4,000 trees ha^-^^1^. Mean diameter at breast height (DBH) and mean height of the trees were 15.9 cm and 16.9 m, respectively. The soil type was Andosol, according to FAO soil classification (Sakai et al., 2010) and there was only sparse understory vegetation, due to the closed canopy (limited light) and litter that covered the forest floor (Konôpka et al., 2006).

In August 2003, 12 plots (1 m × 2 m) were established between planting lines. The plots were fertilized with N from October 2003 to November 2006. Each month, 20 L of 10 mM ammonium nitrate solution was sprayed onto 6 of the 12 plots (336 kg N ha^-^^1^ y^-^^1^). The remaining six plots were supplied with same amount of tap water that was equivalent to 120 mm rainfall y^-^^1^ and used as control plots. Among the 12 established plots, 10 (five fertilized and five control plots) were used for this study. Further information on the site characteristics and the design of the N-fertilization experiment can be found in Noguchi et al. (2013) and Sakai et al. (2010).

### SOIL ANALYSES

Soil coring was conducted in August 2003 (before N fertilization) and November 2006 (19 days after the last N fertilization) using a soil auger with a 4.8 cm inner diameter (Split tube sampler, Eijkelkamp Agrisearch Equipment, Giesbeek, Netherlands). For initial sampling, three core samples were taken at corners of the 1 m × 2 m plots. Each core sample included soil from 0 to 20 cm below the surface. The soil cores were divided into two depth levels (0–10 and 10–20 cm). The soil samples from the three sampling locations in each plot were pooled before air-drying. For final sampling, a soil core sample was taken from the inside of each plot and divided into two depth levels. Thus, one sample of each depth level was obtained for each plot [i.e., *N* = 5 for both the N-fertilized (NF) and the control plots]. Samples were air-dried and sieved through a 2-mm mesh. Roots were removed from the samples. Then, 20 g of each soil sample was suspended in 100 mL of 2 mol L^-^^1^ KCl solution and shaken for 1 h to extract inorganic N. After extraction, the supernatant was clarified by filtration, and the inorganic N concentration was analyzed using flow-injection N analyzers (TN-30 for NH_4_–N and TN-50 for NO_3_–N, Mitsubishi Chemical Analytech, Tokyo, Japan).

### FINE ROOT ANALYSES

Soil coring for fine root analyses were conducted in November 2006 with the same soil auger used for soil analyses. Two 20-cm soil cores were obtained from each plot. The soil cores were divided into two depth levels (0–10 and 10–20 cm) and the soil samples from the two sampling locations in each plot were pooled before root sample preparation. Thus, one sample of each depth level was obtained for each plot (i.e., *N* = 5 for both the NF and the control plots). The soil samples were washed with tap water on a sieve that had a 0.5-mm mesh in order to remove the soil. Fine roots were manually collected using tweezers. Live roots were distinguished from dead roots primarily by root color and resilience, i.e., black and/or easily broken fragile fine roots were assumed to be dead roots (Konôpka et al., 2006, 2007).

The fine roots were divided into diameter classes of <1 mm and 1–2 mm and were subjected to image analysis. The image analysis was conducted using a root image analysis system that included image analysis software and an image scanner (WinRHIZO Pro 2005b, Regent Instruments, Quebec, QC, Canada). To obtain the images, fine root samples were placed in tap water in a transparent plastic tray and were scanned at 400 dpi using the scanner and a transparency unit. Root length, diameter, and volume were used in further analyses. Data were analyzed separately for diameter classes of <0.5, 0.5–1.0, and 1.0–2.0 mm. After the scanning, fine root samples were dried at approximately 70°C for more than 48 h and were weighed.

Specific root length and root tissue density (RTD) were calculated by following equations:

SRL (m g^-^^1^) = fine root length (m m^-^^2^)/fine root biomass (g m^-^^2^) (1)

RTD (g cm^-^^3^) = Fine root biomass (g m^-^^2^)/fine root volume (cm^3^ m^-^^2^) (2)

After weighing, the N concentration of the fine roots was measured using an NC analyzer (Sumigraph NC-22F, SCAS, Tokyo, Japan). Fine roots 1–2 mm in diameter at soil depths of 10–20 cm were not subjected to this N analysis due to limited sample quantity.

### STATISTICS

Split-plot ANOVA was conducted to examine the effects of N fertilization and soil depth on fine root parameters and soil inorganic N contents. Sampling plots were considered as a random effect. Analyses of fine root parameters were performed separately for each fine root diameter class. In addition, one-way ANOVA was conducted to examine effects of N fertilization at each soil depth level. A Wilcoxon rank sum test was performed to examine effects of N fertilization on the proportion of fine root length in different diameter classes. These statistical analyses were performed using JMP 9.0 (SAS Institute, Cary, NC, USA). For the Wilcoxon rank sum test, we referred to a table of critical values due to the small number of replicates.

## RESULTS

### INORGANIC N IN THE SOIL

In August 2003, prior to fertilization, concentrations of NH_4_–N and NO_3_–N were similar in the control and NF plots (**Tables 1 and 2**). In November 2006, 19 days after the final fertilization, NO_3_–N concentrations were significantly greater in the NF plots than in the control plots, but NH_4_–N concentrations did not significantly differ between NF and control plots (**Tables 1 and 2**). The increase in NO_3_–N concentration was more evident in subsurface soil than in the surface soil.

**Table 1:** Concentration of inorganic nitrogen in the soil before and after N fertilization.

	Soil depth (cm)	August 2003		November 2006	
		Control	NF	Control	NF
NH_4_–N (mg kg^-^^1^)	0–10	30 ± 1	31 ± 1	23 ± 3	17 ± 1
	10–20	16 ± 1	15 ± 1	8 ± 0	8 ± 1
NO_3_–N (mg kg^-^^1^)	0–10	12 ± 1	10 ± 2	22 ± 3	39 ± 6
	10–20	16 ± 3	14 ± 3	9 ± 3	127 ± 31

*Data shown are means ± SE (N = 5).*

**Table 2:** Results of split-plot ANOVA examined for effects of N fertilization (treatment) and soil depth (depth) on concentrations of inorganic nitrogen (NH_4_–N and NO^3^–N).

Parameter	Effect	August 2003		November 2006	
		*F*-value	*P*-value	*F*-value	*P*-value
NH_4_–N	Treatment	0.6	0.48	2.3	0.17
	Depth	390	<0.01	66.8	<0.01
	Treatment × depth	1.5	0.25	4.3	0.07
NO_3_–N	Treatment	0.9	0.36	14.3	<0.01
	Depth	5.1	0.05	7.2	0.02
	Treatment × depth	0.0	0.92	12.8	<0.01

### BIOMASS AND LENGTH OF FINE ROOTS

Mean biomass of total fine roots (i.e., fine roots <2 mm in diameter at soil depths of 0–20 cm) was 56 and 88 g m^-^^2^ in the control and the NF plots, respectively. Fine roots <1 mm in diameter accounted for 76% of the total fine root biomass in both plots (**Table 3**). Although mean biomass of the fine roots <1 mm in diameter in the NF plots was 1.6-times as large as that in the control plots, the difference was not statistically significant (**Table 4**). On the other hand, the effect of soil depths on the biomass of fine roots <1 mm in diameter was significant: fine root biomasses in the surface soil were four-times and 2.5-times as large as those in subsurface soil of the control and the NF plots, respectively (**Tables 3 and 4**). The biomass of fine roots 1–2 mm in diameter was not affected significantly by either N fertilization or soil depth (**Table 4**).

**Table 3:** Biomass and length of fine roots of sugi at control and nitrogen-fertilized (NF) plots.

Diameter class	Soil depth (cm)	Biomass (g m^-2^)		Length (m m^-2^)	
		Control	NF	Control	NF
Diameter <1 mm	0–10	34.1 ± 8.0	47.8 ± 10.1	557 ± 146	912 ± 188
	10–20	8.6 ± 5.6	19.4 ± 6.3	125 ± 76	311 ± 100
Diameter 1–2 mm	0–10	12.2 ± 4.1	10.5 ± 3.1	24 ± 7	24 ± 7
	10–20	1.1 ± 0.7	10.8 ± 8.3	6 ± 4	27 ± 17

*Data shown are means ± SE (*N* = 5).*

**Table 4:** Results of split-plot ANOVA examined for effects of N fertilization (treatment) and soil depth (depth) on biomass and length of fine roots.

Parameter	Diameter class	Effect	*F*-value	*P*-value
Biomass	Diameter <1 mm	Treatment	1.8	0.21
		Depth	19.7	<0.01
		Treatment × depth	0.1	0.82
	Diameter 1–2 mm	Treatment	0.4	0.52
		Depth	2.3	0.17
		Treatment × depth	2.5	0.15
Length	Diameter <1 mm	Treatment	3.0	0.12
		Depth	23.1	<0.01
		Treatment × depth	0.6	0.45
	Diameter 1–2 mm	Treatment	0.8	0.39
		Depth	0.8	0.41
		Treatment × depth	1.8	0.22

**FIGURE 1:**
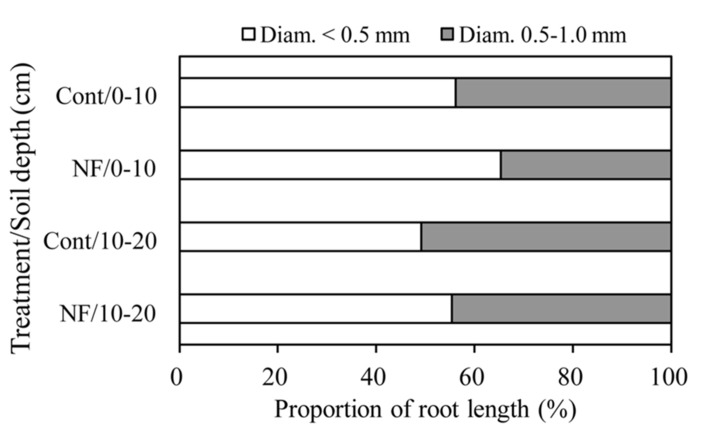
**Proportion of length of fine roots in different diameter classes.** White and gray columns represent fine roots <0.5 mm in diameter and fine roots 0.5–1.0 mm in diameter, respectively.

The mean length of total fine roots (i.e., fine roots <2 mm in diameter at soil depths of 0–20 cm) was 0.71 in control plots and 1.27 km m^-^^2^ in NF plots. Fine roots <1 mm in diameter accounted for 96% of the total fine root length in both plots (**Table 3**). The length of fine roots <1 mm in diameter was significantly larger in the surface soil than in subsurface soil, but the effect of N fertilization on fine root length was not significant (**Table 4**). For fine roots <1 mm in diameter in the surface soil, the proportion of “very” fine roots (diameter <0.5 mm) was significantly greater in the NF plots (65%) than in the control plots (56%; Wilcoxon rank sum test, α = 0.05; **Figure 1**). The length of fine roots 1–2 mm in diameter was not significantly affected by either N fertilization or soil depth (**Table 4**).

### MORPHOLOGY OF FINE ROOTS

The mean diameter of fine roots <1 mm in diameter ranged from 0.52 to 0.57 mm and was significantly smaller in the surface soil than in subsurface soil (**Tables 5 and 6**). In the surface soil, root diameter was marginally smaller in the NF plots than in the control plots (**Table 5**; One-way ANOVA, *P* = 0.06). Mean RTD of fine roots <1 mm in diameter ranged from 0.23 to 0.26 g cm^-^^3^ and was not significantly affected by either soil depth or N fertilization (**Tables 5 and 6**). Mean SRL of fine roots <1 mm in diameter ranged from 16.0 to 19.2 m g^-^^1^ (**Table 5**). Although the effects of soil depth and N fertilization on SRL were not significant, their interactive effect was marginally significant (*P* = 0.05; **Table 6**), indicating that N fertilization affected SRL differently at different soil depths. At soil depths of 0–10 cm, SRL was significantly greater in NF plots than in control plots (One-way ANOVA, *P* = 0.01). SRL did not differ significantly between NF and control plots at soil depths of 10–20 cm.

**Table 5:** Morphological parameters of fine roots of sugi at control and nitrogen-fertilized (NF) plots.

Diameter class	Soil depth (cm)	Diameter (mm)		RTD (g cm^-3^)		SRL (m g^-1^)	
		Control	NF	Control	NF	Control	NF
Diameter <1 mm	0–10	0.55 ± 0.02	0.52 ± 0.01	0.26 ± 0.01	0.25 ± 0.01	16.0 ± 0.9	19.2 ± 0.3
	10–20	0.57 ± 0.03	0.57 ± 0.01	0.23 ± 0.01	0.24 ± 0.01	17.9 ± 2.1	16.1 ± 0.5
Diameter 1–2 mm	0–10	1.4 ± 0.1	1.3 ± 0.1	0.33 ± 0.03	0.33 ± 0.03	2.2 ± 0.4	2.3 ± 0.3
	10–20	1.3^a^	1.2 ± 0.1	0.17^a^	0.20 ± 0.07	5.1^a^	7.0 ± 3.0

*Data shown are means ± SE (N = 2–5).*

a Standard error (SE) was not shown because the number of replication was 2 for these data.

**Table 6:** Results of split-plot ANOVA examined for effects of the N fertilization (treatment) and soil depth (depth) on diameter, root tissue density (RTD) and specific root length (SRL) of fine roots <1 mm in diameter.

Parameter	Effect	*F*-value	*P*-value
Diameter	Treatment	0.8	0.41
	Depth	11.5	0.01
	Treatment × depth	3.7	0.09
RTD	Treatment	0.0	0.90
	Depth	3.4	0.10
	Treatment × depth	1.7	0.23
SRL	Treatment	0.3	0.62
	Depth	0.3	0.60
	Treatment × depth	5.2	0.05

The diameter, RTD, and SRL of fine roots 1–2 mm in diameter in the surface soil were similar in control and NF plots and were not significantly different. Statistical analyses were not performed for the fine roots 1–2 mm in diameter in the subsurface soil, because there were no fine roots in this diameter class in the three of five control samples.

### N CONCENTRATION IN FINE ROOTS

Nitrogen concentration ranged from 16.2 to 18.2 g kg^-^^1^ in fine roots <1 mm in diameter, whereas it ranged from 7.7 to 9.0 in roots that were 1–2 mm in diameter (**Table 7**). The N concentration of fine roots <1 mm in diameter in the surface soil was marginally higher in NF plots than in control plots (One-way ANOVA, *P* = 0.06). However, there was no significant difference between control and NF plots in the N concentration of roots in the subsurface soil or the N concentration of roots 1–2 mm in diameter (data not shown). The effects of soil depth on N concentrations in fine roots were also not significant (data not shown).

**Table 7:** N concentrations in fine roots of sugi in control and nitrogen-fertilized (NF) plots.

Diameter class	Soil depth (cm)	N concentration (g kg^-1^)	
		Control	NF
Diameter <1 mm	0–10	16.6 ± 0.5	18.2 ± 0.5
	10–20	17.9 ± 1.1	16.2 ± 1.1
Diameter 1–2 mm	0–10	7.7 ± 0.6	9.0 ± 0.6
	10–20	N.A.	N.A.

*Data shown are means ± SE (*N* = 4–5).*

*N.A., not analyzed.*

## DISCUSSION

### EFFECTS OF N FERTILIZATION ON THE SOIL

After the 3 years of N fertilization, there was a greater concentration of NO_3_–N in the soil of NF plots than in control plots, whereas NH_4_–N concentration was not significantly different between the plots. Soil pH decreased in NF plots (from 5.5 to 4.5; Noguchi et al., 2013) and this, when considered together with the results of the present study, suggests that fertilization facilitated nitrification. In addition, the differences in NO_3_–N concentration between NF and control plots were more evident in the subsurface soil than in the surface soil. This vertical gradient of NO_3_–N in the soil can be attributed to downward movement of ${NO}_{3}^{-}$ ions due to their high mobility, which was often linked to N export from forested watersheds (Ohrui and Mitchell, 1997; Shibata et al., 2001).

Nagakura et al. (2006) conducted a 7-year N-fertilization experiment in an adjacent sugi stand. In that study, NH_4_NO_3_ solution was supplied at the same rate as in the present study (336 kg N ha^-^^1^ y^-^^1^). They found that N fertilization increased ${NO}_{3}^{-}$ concentration and decreased pH in the soil solution, which is consistent with our results. In addition, they reported that concentrations of Ca^2^^+^ and Mg^2^^+^ in the soil solution also increased after 2–3 years of fertilization. Thus, cations might also have been released in the NF plots during the present study.

### EFFECTS OF N FERTILIZATION ON FINE ROOTS

A number of studies have indicated that soil fertility affects fine root biomass (Yuan and Chen, 2010). In natural conditions, for example, fine root biomass or biomass allocation to fine roots decreased with increasing N availability along slopes (Tateno et al., 2004) or latitudinal gradients (Helmisaari et al., 2007). On the other hand, N fertilization may increase, decrease, or have no effect on fine root biomass. In Norway spruce (*Picea abies*) stands in Finland and Sweden, for example, N fertilization increased fine root biomass, except at a site that had a fertile soil type (Helmisaari and Hallbäcken, 1999). In contrast, N fertilization combined with wood ash application tended to decrease fine root biomass (Helmisaari et al., 2009). These results suggest that soil fertility together with other chemical conditions in the soil affect the responses of fine roots to N fertilization. The scale of fertilization also affects the pattern of changes in fine root biomass. For example, N fertilization of micro-sites often results in the proliferation of fine roots (Pregitzer et al., 1993; Hodge, 2004).

In the present study, we showed that the biomass and length of fine roots appeared to be larger in NF plots than in control plots, although the difference between the plots was not significant (**Tables 3 and 4**). A previous study at the same study site indicated that the elongation rate of fine roots <1 mm in diameter was higher and the residence time of the fine roots was longer in NF plots than in control plots (Noguchi et al., 2013). Thus, in the present study, the sugi trees likely responded positively to N fertilization by increasing the biomass and length of fine roots.

The effects of N fertilization were more evident in fine root morphology than in fine root biomass. We found that the 3 years of N fertilization significantly increased the SRL of fine roots <1 mm in diameter in the surface soil (**Table 5**). The SRL can be described as a function of the diameter and RTD of fine roots (Ostonen et al., 2007b). Our results showed that diameter of fine roots <1 mm in diameter in the surface soil was marginally smaller in NF plots than in the control plots (0.52 vs. 0.55 mm, **Table 5**), whereas there was no effect of N fertilization on the RTD of these fine roots (**Tables 5 and 6**). Furthermore, in the surface soil, the proportion (by length) of “very” fine roots (diameter <0.5 mm) was significantly greater in the NF plots than in the control plots (**Figure 1**). These results suggest that the N fertilization in this study increased the amount of thinner fine roots relative to the amount of thicker fine roots or increased branching of fine roots. Branching of fine roots can result from proliferation of lower-order roots with small diameter (Pregitzer et al., 2002).

The SRL of fine roots has been previously reported to vary with soil N conditions. A meta-analysis by Ostonen et al. (2007b) indicated that the SRL of fine roots generally decreases with N fertilization. This was also supported by a recent study, in which N fertilization decreased SRL of *Pinus tabuliformis* roots, especially for 1st- and 2nd-order roots (Wang et al., 2013). On the other hand, under heterogeneous soil nutrient conditions, root proliferation often occurs in nutrient rich patches and rapid root proliferation is linked to high SRL (Eissenstat, 1991; Hodge, 2004). SRL has often been used as an index of the cost and benefit of fine roots, assuming that root length is proportional to resource acquisition (benefit) and root mass is proportional to construction and maintenance (cost; Eissenstat and Yanai, 1997). In addition, SRL is reported to be positively correlated with root respiration and N uptake (Reich et al., 1998; Makita et al., 2009). Therefore, the increased SRL of fine roots <1 mm in diameter that was observed in the present study may be a cost-effective response of sugi to increase acquisition of soil resources from N-rich sites.

It is, however, still difficult to explain why SRL increased in the NF plots only in the surface soil and not in the subsurface soil (**Table 5**). Increased N availability is not the only reason because the concentration of inorganic N was much greater in the subsurface soil than in the surface soil (**Table 1**). This vertical pattern of N concentration in the soil is also not consistent with the vertical pattern of N concentrations in fine roots (**Table 7**). In the NF plots, most of inorganic N in the subsurface soil was present as ${NO}_{3}^{-}$ that was highly mobile in the soil solution (**Table 1**). In this condition, roots might not need to proliferate to increase the N acquisition (Hodge, 2004). A recent study of four Norway spruce stands suggested that vertical patterns of SRL might be associated with a base-saturation gradient (Borken et al., 2007). In our study site, N fertilization likely increased cation release into the soil solution, as occurred in an adjacent sugi stand (Nagakura et al., 2006). Soil physical properties, such as porosity and bulk density, might also affect SRL, as mentioned by Borken et al. (2007). These chemical and physical characteristics of the soil may also affect the morphological responses of fine roots to N fertilization observed in this study.

In conclusion, this study suggests that the effect of high N load was more evident in fine root morphology than in fine root biomass. Fine root biomass tended to increase under high N load, although the effect was not statistically significant. The increase in SRL in the surface soil may reflect a cost-effective means of acquiring soil resources from N-rich patches. However, the observed differences in SRL could not be fully explained by increased N availability, given their vertical patterns. A better understanding of sugi responses to high N load may be gained from future studies in which chemical and physical characteristics of the soil, such as cation leaching or porosity, are examined together with fine root characteristics.

## Conflict of Interest Statement

The authors declare that the research was conducted in the absence of any commercial or financial relationships that could be construed as a potential conflict of interest.
